# Abstract of Report of the State Veterinary Surgeon of Montana

**Published:** 1903-02

**Authors:** M. E. Knowles

**Affiliations:** State Veterinarian


### ABSTRACT OF REPORT OF THE STATE VETERINARY SURGEON FOR MONTANA.

THE success and prosperity of the live-stock industry of a commonwealth depends largely upon the healthfulness of its domestic animals, and the general condition of the domestic animals of Montana to-day from a health point of view is most satisfactory. There are a number of factors conducive to this most happy condition in our State, one among which is the existence of the major portion of our domestic animals under perfectly natural conditions—that is, the major portion of the domestic animals of Montana live an all-out-of-door life that is conducive to good health and strong constitution. The other important factor having a decidedly direct influence in this regard is our most excellent live-stock sanitary laws, the intelligence of our live-stock growers, and the personal interest taken by our live-stock growers in enforcing sanitary regulations. Experience and carefully conducted scientific experiments have shown that disease-producing germs or organisms cannot live or thrive in bright sunlight. It is not difficult, therefore, under ordinary range conditions, if a contagious disease is introduced to manage and repress it by carefully enforced sanitary regulations. However, conditions are changing in our State, and within a few years a majority of our domestic animals will be under a much closer system of domestication. The open range will give way to fenced pastures, the corral and shed will be superseded by barns and stables. The native hay will be supplanted by cultivated grasses, the cattle whip will take the place of the lasso, and a complete change will necessarily occur in the handling of all our domestic animals. It is important, therefore, that this change should occur with due regard for sanitary surroundings and the prevention of diseases. In crowded cities or closely settled rural districts where domestication is practised in its full meaning, all diseases are usually more frequent than they are under perfectly natural conditions. Unsanitary stables, improper food, and generally bad hygienic surroundings are conducive to the development of non-contagious and non-infectious diseases as well as in the propagation of devastating contagious and infectious animal diseases. Accidents and non-contagious and non-infectious animal diseases are occurring with more or less frequency in all communities, and it is, therefore, our duty to acquire and utilize all the knowledge possible toward the prevention of diseases. Therefore, as our conditions change in Montana, it is to be hoped our live-stock growers will take cognizance of these facts and in the construction of their stables and sheds, give careful consideration to light, ventilation and drainage. The dark, badly drained, unsanitary stables of the East and in Continental countries have been responsible for the rapid development of tuberculosis among cattle and other diseases inimical to public health. The education and intelligence of the stock-grower is the most potent factor in the prevention and suppression of contagious or infectious animal diseases, and Montana is most fortunate indeed in having one of the most intelligent stock-growing communities in the world, therefore one of the freest, if not the freest, State in the Union from contagious and infectious animal diseases, and it should be our aim to so maintain it. The great majority of stock-growers throughout Montana take a personal interest in the healthfulness of our domestic animals, and contrary to the custom obtaining in other States where nearly all reports of contagious or infectious disease are made by neighbors or others acquainted with the suspected fact, in Montana it is almost invariably the rule that the owner personally makes such report and is the most anxious of all to have the matter investigated, and is the most active one in assisting by every means within his power in its repression.

This distribution of blackleg vaccine has saved our cattlemen many thousands of dollars annually. In fact, figuring the loss at 2 per cent., which is a very low estimate, the free distribution of vaccine is saving our cattle interest at least $40,000 per annum in loss from this disease. Vaccination against blackleg has become so universal throughout Montana, that it is safe to say fully 90 per cent. of our cattlemen are familiar with the operation and are successfully employing it every year.

*Tuberculosis.* Fortunately, so far, we have but little tuberculosis in our State. I believe that I can safely say there is no tuberculosis among range cattle. There is probably some tuberculosis among dairy herds throughout the State, but so far it has attracted but little, if any, attention from owners if it does exist, and but few cases have been discovered, only eight animals being destroyed on account of tuberculosis during the year. The important thing for us to do is to endeavor to keep Montana free from this animal scourge, which we can certainly do by the strict enforcement of rigid quarantine regulations and by insisting upon a health certificate, including tuberculin test for all breeding or dairy cattle shipped into the State. I regard as the greatest menace to our general cattle industry the possibility of the existence of tuberculosis among the dairy herds of our State; but it is an utter impossibility for this office to test all the dairy herds in Montana without considerable additional assistance. In my opinion, which is based upon general observation, the dairy herds of Montana are comparatively free from this source. In any event, if the disease exists, it has not attracted sufficient attention to demand investigation; however, tuberculosis among cattle in many instances is such an insidious disease, that it does not attract the attention of the laymen. Municipal meat- and milk-inspection must ultimately be adopted in our State, and its inauguration and maintenance under competent administration will not alone be of inestimable value from a public health standpoint, but will materially lessen the possibility of serious infectious or contagious disease to our beef herds.

Prof. Behring, of the University of Berlin, has recently published a preliminary report of his antitoxin against tuberculosis and has shown conclusively that he has an antitoxin that is efficacious. His experiments during the past four or five years have shown that this antitoxin has sufficient immunizing power to protect cattle against artificial attempts at infection; this being the case, it is safe to assume that this antitoxin will certainly protect against the natural methods of infection, which are less direct and have less potent powers of transmission than the artificial means. The perfection of this antitoxin and its universal employment means more to the cattle industry from an economical standpoint than any discovery that has been made in the medical world.

*Blackleg.* Blackleg vaccination has become so general throughout Montana that the loss from this disease is comparatively insignificant. There have been but few reports of losses from blackleg during the past year. All of these have been investigated, and in every instance the percentage of loss was less than one-half of 1 per cent. If vaccination against blackleg increases in popularity as it has during the past five years, within another ten years it is pretty safe to assume that blackleg will be an unknown disease in Montana.

*Glanders.* During the past year eighty-three horses have been destroyed on account of glanders, and 1593 placed in quarantine on account of being exposed to this disease. Glanders should be an unknown disease within our borders, and it would be, were it not for the carelessness of many of our owners in constantly keeping a horse suffering from this disease in contact with his healthy horses. I have endeavored to impress upon our horse-owners the importance of isolating all suspected horses until such time as they are proved free from this most serious of all horse diseases, and I again desire to call the attention of our horse interests to this important matter. Whenever a horse is found suffering with a chronic discharge from the nose, he should be at once isolated and kept entirely away from all other horses until he has been examined by a competent local veterinarian or one from this office. If this procedure is faithfully carried out, there will be but little trouble in ridding our State of glanders.

*So-called Montana Horse Disease, Swamp Fever, Malaria of the Horse or Mountain Fever.* This disease has occurred among possibly fifty head of horses in various localities throughout the State during the past year. In the Red River Valley of the North, the same disease obtains and is more destructive in that locality than it is with us. Recently some investigations have been carried on relative to this disease in the Minnesota State Bacteriological Laboratory. The results of this investigation have not as yet been given out, but it is claimed that they have discovered a micro-organism to which the disease is due. In years past this disease has been very destructive in different localities in Montana, a few owners having met with very great losses. One of the heaviest losses reported to me, which occurred some time during the early nineties, was among a band of horses owned in Meagher County, in which it was claimed, I believe, that about 500 perished. Did the disease continue to exist in this manner or reappear so as to occasion such serious losses, it would be a most serious affair. In many respects the so-called Montana Horse Disease resembles closely an Asiatic disease known as "surra;" however, they are not identical. The "Montana Horse Disease" always occurs in swampy localities, and I have regarded it as being due to some micro-organisms obtained through the medium of the food, or some animal parasites picked up by horses in these swampy areas. Investigation will ultimately prove the truth of this conjecture. In any event, the disease is of sufficient economical importance to warrant its careful study.

*Contagious Abortion among Cattle.* Contagious abortion among cattle has occurred to some considerable extent in a number of localities throughout the State during the past year. This is a most disastrous disease and one exceedingly difficult to deal with under the most favorable conditions. It is particularly difficult to manage under range conditions.

However, where cattle are under close domestication, it can be controlled by careful attention to ordinary sanitary measures, such as the thorough disinfection of stables and sheds, by disinfecting the bull and keeping him away from cows for a few weeks, and by injecting the exposed cows two or three times a week with 2 c.c. of a 5 per cent. solution of carbolic acid, this injection to be given hypodermically with the ordinary blackleg vaccine syringe. The bull's penis should be washed with a weak solution of creolin, the sheath injected with the same solution, and all the lower portion of the belly be carefully washed in this solution once every day or two for at least a week. All manure and litter of all kinds should be cleaned out of the sheds and stables, spread out in the sunlight to dry, and then be carefully burned. Sheds and stables should be thoroughly whitewashed with whitewash containing at least 2 per cent. of carbolic acid, and where possible sheds should have roofs removed so as to permit the sunlight for several weeks to do its part in disinfecting the interior. Stables should be as thoroughly aired as possible after disinfection and all the sunlight permitted to enter that is possible.

*Contagious Ophthalmia or Pink Eye of Cattle So-called.* This disease of the eyes of the cattle is too well-known throughout the State to warrant description, as it has appeared very generally all over the State during the past three or four years. It is impossible to treat range cattle, but, fortunately, the disease is not destructive to the sight, excepting in a very small percentage of cases. It is true that during the continuance of the disease, the animal loses condition rapidly, and some of them, where both eyes are affected, if away from water, are likely to suffer considerably from thirst and consequent loss of condition. The disease during its continuance is evidently quite painful, and the majority of thus affected droves of cattle do not thrive during its continuance, but within from two to three weeks recover their normal condition, with a very small percentage losing one eye. It is rare indeed that both eyes are destroyed. Domesticated cattle can be materially benefited by treatment. It is advisable in cattle that can be handled, on the appearance of the disease, to at once separate the diseased from the healthy, and to the diseased administer a dose of Epsom salts—to an adult, from one pound to one and one-half pounds; proportionately smaller doses for younger cattle. Bathe the affected eyes two or three times a day in salt-water solution, one teaspoonful of salt to each quart of water that has been thoroughly boiled and allowed to cool. In addition to this, a few drops of the following solution in the eyes twice a day will be found of benefit: sulphate of zinc, 2 grains; sulphate of atropia, 4 grains; and distilled water, 1 oz.; mixture. An eye-dropper half-full injected into each eye morning and night. It is of importance, where possible, to keep these animals confined in a dark shed or stable, as the light is extremely painful to them.

*Hog Cholera.* Hog cholera has appeared in three different localities in the State during the past year, and in every instance the infection was believed to have been traced to the railroads. Many hogs are shipped into Montana annually from Eastern States in which cholera constantly exists, and occasionally hogs develop cholera after being loaded into the cars. The débris from these cars shaking out along the right-of-way and into streams as the cars pass over bridges, has been responsible for nearly all of our infections.

Railroad companies should be compelled to disinfect all cars in which swine are transported after each shipment. In fact, all cars used for the transportation of any domestic animals should be so treated.

*Poisonous Plants.* Poisonous plants also contribute their share in occasioning losses among our domestic animals. During the past year losses from this source have been investigated among cattle, the most serious being due to lupine and water-hemlock. It is impossible to guard against these losses under range conditions, but where cattle are herded or pastured, the affected localities can be avoided, or, if not too extensive in area, can be fenced off so that they are inaccessible to animals. When animals suffering from plant poisoning are found in time, the administration of from ten to fifteen grains of permanganate of potassium, dissolved in from one pint to one quart of water, and repeated once or twice at an interval of an hour or two, will usually cure. It is always well after the animal has recovered from the acute effects of the poisoning, to administer a physic of Epsom salts or raw linseed oil. In the case of cattle, administer from one pound to one and a half pounds of Epsom salts, and of raw linseed oil from one quart to a half gallon.

*Kaffir Corn Poison.* As Kaffir corn is an arid land forage likely to be quite generally used in Montana as a cattle food, it is important our cattle-growers should understand the apparent danger of pasturing this plant in its green state. Kaffir corn, it is maintained by Prof. Avery, of the Nebraska Experiment Station, contains a large percentage of prussic acid, and when cattle are pastured upon it in its green stage, they are very prone to the poison. One instance is reported in Colorado where twenty-five dairy cows out of thirty-one became sick within five minutes after they were turned into a patch of Kaffir corn, and within two hours, twenty-five of them were dead. Kaffir corn is said to be an excellent forage for cattle when cured, but it seems, from recent experiments, it is extremely dangerous in its green state.

During the year ending October 1, 1902, the number of horses inspected for glanders was 1673; number quarantined, 1593; destroyed, 83; health certificates issued, 1102; amount of money expended for burial fees, $415.00.

Number of cattle inspected, 15,602, of which 506 were for tuberculosis; 300 for blackleg; 4,200 for ophthalmia; 600 for scab; quarantined for tuberculosis, 44; quarantined for scab, 600; destroyed for tuberculosis, 8; health certificates issued, 5626; money expended for burial fees on account of tuberculosis, $40.

Number of sheep inspected for scab, ictero hæmaturia, and plant poisoning, 171,287; quarantined, 144,125; health certificates issued, 788.

There were 30,295 doses of blackleg vaccine distributed to 306 persons in the State during the year.

Number of Southern cattle for which health certificates were received, 112,567.

M. E. Knowles,
State Veterinarian.

